# Radiotherapy in localized prostate cancer: a multicenter analysis evaluating tumor control and late toxicity after brachytherapy and external beam radiotherapy in 1293 patients

**DOI:** 10.1007/s00066-024-02222-w

**Published:** 2024-03-15

**Authors:** Matthias Moll, Elisabeth Nechvile, Christian Kirisits, Oxana Komina, Thomas Pajer, Bettina Kohl, Marcin Miszczyk, Joachim Widder, Tomas-Hendrik Knocke-Abulesz, Gregor Goldner

**Affiliations:** 1 Department of Radiation Oncology, Comprehensive Cancer Center, Medical University of Vienna, Vienna, Austria; 2 Department of Radiation Oncology, Klinik Hietzing, Vienna, Austria; 3 IIIrd, Maria Skłodowska-Curie National Research Institute of Oncology, Wybrzeże Armii Krajowej 15, 44-102 Gliwice, Poland; 4 Department of Radiation Oncology, Medical University of Vienna, Währinger Gürtel 18–20, 1090 Vienna, Austria

**Keywords:** Low-risk, Intermediate-risk, Austria, HDR, LDR

## Abstract

**Background and purpose:**

Comparing oncological outcomes and toxicity after primary treatment of localized prostate cancer using HDR- or LDR-mono-brachytherapy (BT), or conventionally (CF) or moderately hypofractionated (HF) external beam radiotherapy.

**Materials and methods:**

Retrospectively, patients with low- (LR) or favorable intermediate-risk (IR) prostate cancer treated between 03/2000 and 09/2022 in two centers were included. Treatment was performed using either CF with total doses between 74 and 78 Gy, HF with 2.4–2.6 Gy per fraction in 30 fractions, or LDR- or HDR-BT. Biochemical control (BC) according to the Phoenix criteria, and late gastrointestinal (GI), and genitourinary (GU) toxicity according to RTOG/EORTC criteria were assessed.

**Results:**

We identified 1293 patients, 697 with LR and 596 with IR prostate cancer. Of these, 470, 182, 480, and 161 were treated with CF, HF, LDR-BT, and HDR-BT, respectively. For BC, we did not find a significant difference between treatments in LR and IR (*p* = 0.31 and 0.72). The 5‑year BC for LR was between 93 and 95% for all treatment types. For IR, BC was between 88% in the CF and 94% in the HF group. For CF and HF, maximum GI and GU toxicity grade ≥ 2 was between 22 and 27%. For LDR-BT, we observed 67% grade ≥ 2 GU toxicity. Maximum GI grade ≥ 2 toxicity was 9%. For HDR-BT, we observed 1% GI grade ≥ 2 toxicity and 19% GU grade ≥ 2 toxicity.

**Conclusion:**

All types of therapy were effective and well received. HDR-BT caused the least late toxicities, especially GI.

**Supplementary Information:**

The online version of this article (10.1007/s00066-024-02222-w) contains supplementary material, which is available to authorized users.

## Introduction

Prostate cancer is an age-related disease [1]. Being the most common cancer in men [1] in an aging society, more cases are to be expected and more resources for treatment are required. For localized prostate cancer, 3 different options for approaching the primary tumor exist. These are active surveillance, radical prostatectomy, and radiotherapy. All options achieve the same results regarding overall survival [2]. Active surveillance is often discontinued in clinical practice [3, 4], increasing the relevance of the other two options.

Focusing on radiotherapy, today’s treatment options for primary localized prostate cancer are either external beam radiotherapy (EBRT) in conventional, moderate, or ultrahypofractionation, LDR- and HDR-brachytherapy (BT) as a monotherapy, or a combination of EBRT and BT [5]. While these are assumed to be equally effective, differences are reported regarding toxicity [6]. However, to our knowledge, there is no study comparing all these approaches at once.

Therefore, we want to provide an overview of tumor control, as well as late gastrointestinal (GI) and genitourinary (GU) toxicity, after treatment with conventionally (CF) or moderately hypofractionated (HF) EBRT, or LDR- or HDR-mono-BT in primary localized prostate cancer as a bicenter study.

## Materials and methods

The study protocol was approved by the local ethics committees according to local laws and regulations. Patients were retrospectively included in two Viennese departments of radiation oncology during 03/2000 and 09/2022. All patients were treated locally, using CF, HF, or LDR- or HDR-mono-brachytherapy. Both centers offer EBRT, and one offers HDR- and the other LDR-brachytherapy. However, one center provided only data for HDR-mono-brachytherapy. The options of EBRT and brachytherapy were discussed with all patients. The final treatment decision was left to the patient. All patients were considered either low or intermediate risk according to the NCCN guidelines [5], and staged cNX/0 and cMX/0. For EBRT, all patients were treated using either 3D-conformal radiotherapy, intensity-modulated radiotherapy, or the volumetric modulated arc technique, depending on the standard of care at that time. EBRT doses ranged between 74 and 82.1 Gy EQD2, assuming an α/β of 1.5 Gy. Doses were prescribed according to ICRU 50, 62, and 83 [7–9]. The CTV encompassed the prostate in low-risk patients. For patients with intermediate risk, the base of the seminal vesicles was included. Safety margins were 5–10 mm for patients with gold fiducial markers and 7–10 mm for patients without. All patients were treated in supine position. If the treatment was performed primarily, a rectal balloon was used [10]. All patients with a dose of at least 2.25 Gy per fraction were considered moderately hypofractionated.

Before brachytherapy, a preplanning ultrasound was performed. For LDR-BT, I‑125 seeds were transperineally implanted as a monotherapy, using spinal anesthesia. Patients stayed in hospital for 3 days. Dose prescription was 145 Gy to the prostate according to the TG137 protocol [11]. The source strength was on average 0.57 µGy × m^2^/h per seed. For HDR-BT as monotherapy, patients received 3 or 4 fractions of either 9 or 10.5 Gy per fraction. Implantations were performed according to the GEC/ESTRO recommendations available at the time of treatment [12, 13].

Clinical controls were performed during therapy, if requested by the patient, directly after therapy, after 6 weeks, if brachytherapy was performed, 3 months, 12 months, and every year from then on. From the 3‑month follow-up on, the PSA value was measured. A nadir +2 µg/L was considered a biochemical recurrence, according to the Phoenix criteria [14]. GI and GU toxicity were compiled at every clinical control, using RTOG criteria [15].

Statistical analysis was performed using GraphPad Prism 9.5.1 (GraphPad Software, San Diego, CA, USA) and SPSS 28.0.1.1 (IBM, Armonk, NY, USA). We considered a *p*-value < 0.05 as statistically significant. Biochemical control was compared using the Kaplan-Meier method and the log-rank test. For comparison of toxicities, we used the Kruskal-Wallis test. Uni- and multivariable analyses were performed for biochemical control using the treatment type (dichotomized values), the initial PSA (continuous values), ADT duration (continuous values), and age at therapy (continuous values) for low-risk (LR) and intermediate-risk (IR) patients, and the treatment type, For IR patients, we also included Gleason Score (dichotomized values) and T category (dichotomized values).

## Results

In total, we were able to identify 1293 patients with LR and IR prostate cancer in accord with our inclusion criteria. All patients receiving conventionally fractionated EBRT (CF), moderately hypofractionated EBRT (HF), and LDR-BT were treated at one center. All patients treated with HDR-BT were treated at the other center. A detailed breakdown of patient characteristics can be found in Table 1, 2 and 3. A detailed list of prescribed doses and distribution among patients can be found in Supplement 1.

**Table 1:** Patient characteristics for the whole collective

All patients	CF	%	HF	%	LDR-BT	%	HDR-BT	%
*n*	470	100	182	100	480	100	161	100
*T category*								
T1a-c/T2a	383	81	159	87	453	94	143	89
T2b/c	87	19	23	13	27	6	18	11
*Gleason Score*								
≤ 6	374	80	112	62	389	81	108	67
7	96	20	70	38	91	19	53	33
Median initial PSA (IQR) in µg/L	7.0 (5.5/9.8)	–	7.0 (5.5/9.4)	–	6.3 (5.2/8.1)	–	6.4 (5.2/7.6)	–
*Risk group*								
Low risk	226	48	72	40	316	66	83	52
Intermediate risk	244	52	110	60	164	34	78	48
Median age at therapy (IQR) in years	72 (66/75)	–	73 (69/77)	–	69 (63/74)	–	66 (61/72)	–
ADT administered	194	41	50	27	81	17	1	1
Median duration of ADT (IQR), if applied, in months	8 (6/15)	–	6 (6/12)	–	6 (4/9)	–	6 (6/6)	–
Median follow-up (IQR, min, max) in months	84 (48/121, 3, 239)	–	48 (24/60, 3, 84)	–	72 (36/117, 3, 244)	–	59 (37/82, 3, 124)	–
Treatment using 3D-conformal RT	400	85	0	0	–			
Treatment using IMRT or VMAT	70	15	182	100	–			

*ADT* androgen deprivation therapy, *EBRT* external beam radiotherapy, *RT* radiotherapy, *IMRT* intensity-modulated radiotherapy, *VMAT* volumetric modulated arc therapy, *BT* brachytherapy, *min* minimum, *max* maximum

**Table 2:** Patient characteristics for patients with low-risk prostate cancer

Low risk	CF	%	HF	%	LDR-BT	%	HDR-BT	%
*n*	226	100	72	100	316	100	83	100
*T category*								
T1a-c/T2a	226	100	72	100	316	100	83	100
*Gleason Score*								
≤ 6	226	100	72	100	316	100	83	100
Median initial PSA (IQR) in µg/L	6.5 (5.2/8.0)	–	7.1 (5.8/8.0)	–	6.3 (5.3/7.9)	–	6.4 (5.0/7.6)	–
Median age at therapy (IQR) in years	70 (65/74)	–	73 (68/77)	–	69 (63/74)	–	66 (60/71)	–
ADT administered	77	34	9	13	45	14	0	0
Median duration of ADT (IQR), if applied, in months	6 (6/11)	–	6 (3/6)	–	5 (3/8)	–		
Median follow-up (IQR, min, max) in months	86 (48/132, 3, 239)	–	48 (24/60, 3, 84)	–	73 (42/120, 3, 242)	–	65 (38/88, 5, 118)	–
Treatment using 3D-conformal RT	197	87	0	0	–			
Treatment using IMRT or VMAT	29	13	72	100	–			

*ADT* androgen deprivation therapy, *EBRT* external beam radiotherapy, *RT* radiotherapy, *IMRT* intensity-modulated radiotherapy, *VMAT* volumetric modulated arc therapy, *BT* brachytherapy, *min* minimum, *max* maximum

**Table 3:** Patient characteristics for patients with intermediate-risk prostate cancer

Intermediate risk	CF	%	HF	%	LDR-BT	%	HDR-BT	%
*n*	244	100	110	100	164	100	78	100
*T category*								
T1a-c/T2a	157	64	87	79	137	84	60	77
T2b/c	87	36	23	21	27	16	18	23
*Gleason Score*								
≤ 6	148	61	40	36	73	45	25	32
7	96	39	70	64	91	55	53	68
Median initial PSA (IQR) in µg/L	7.4 (5.8/12.1)	–	6.9 (5.4/11.1)	–	6.5 (5.2/10.7)	–	6.4 (5.3/7.6)	–
Median age at therapy (IQR) in years	73 (68/76)	–	74 (69/77)	–	69 (63/75)	–	66 (61/73)	–
ADT administered	117	48	41	37	36	22	1	1
Median duration of ADT (IQR), if applied, in months	9 (6/24)	–	7 (6/14)	–	6 (6/10)	–	6 (6/6)	–
Median follow-up (IQR, min, max) in months	78 (44/108, 3, 226)	–	47 (25/55, 3, 84)	–	62 (32/108, 3, 244)	–	50 (35/76, 3, 124)	–
Treatment using 3D-conformal RT	203	83	0	0	–			
Treatment using IMRT or VMAT	41	17	110	100	–			

*ADT* androgen deprivation therapy, *EBRT* external beam radiotherapy, *RT* radiotherapy, *IMRT* intensity-modulated radiotherapy, *VMAT* volumetric modulated arc therapy, *BT* brachytherapy, *min* minimum, *max* maximum

**Fig. 1:**
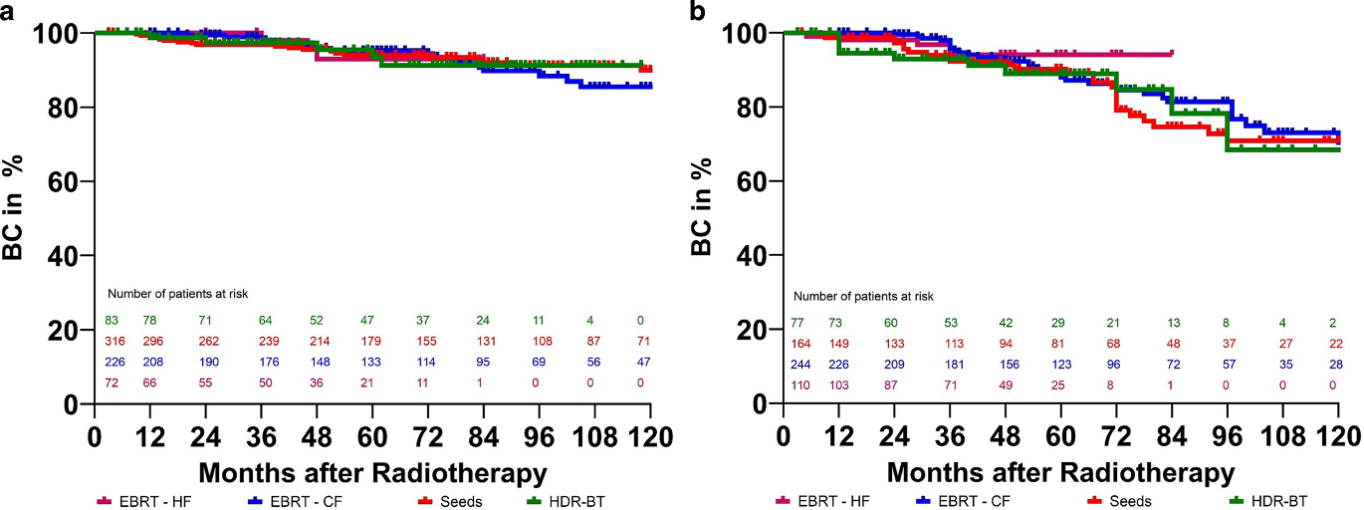
**a** Biochemical control (BC) in patients with low-risk prostate cancer after treatment with HDR brachytherapy, LDR brachytherapy (seeds), conventional (CF), or moderately hypofractionated (HF) external beam radiotherapy. *P* = 0.31, **b** Biochemical control (BC) in patients with intermediate-risk prostate cancer after treatment with HDR brachytherapy, LDR brachytherapy (seeds), conventional (CF), or moderately hypofractionated (HF) external beam radiotherapy. *P* = 0.72

Furthermore, we analyzed biochemical control. The results can be found in Fig. 1a, b. We did not observe any significant differences between groups. For patients with low-risk prostate cancer, the respective BC rates after 5 years were 95%, 93%, 94%, and 93% for CF, HF, LDR-BT, and HDR-BT, and after 10 years were 85%, 90%, and 91% for CF, LDR-BT, and HDR-BT. The respective BC rates for patients with intermediate-risk prostate cancer after 5 years were 88%, 94%, 90%, and 89% for CF, HF, LDR-BT, and HDR-BT, and after 10 years were 70%, 71%, and 68% for CF, LDR-BT, and HDR-BT. No 10-year data for HF in either risk group were available. We also performed uni- and multivariable analyses regarding biochemical control in LR and IR. The results can be found in Tables 4 and 5.

**Table 4:** Uni- and multivariable analysis of patients with low-risk prostate cancer regarding biochemical control

Low risk	Univariable analysis				Multivariable analysis			
Parameter	HR	95% CI (lower bound)	95% CI (upper bound)	*p*-value	HR	95% CI (lower bound)	95% CI (upper bound)	*p*-value
**Type of therapy (CF baseline)**								
Vs. HF	1.012	0.292	3.504	0.986	0.825	0.236	2.881	0.763
Vs. LDR	0.845	0.453	1.577	0.596	0.692	0.364	1.315	0.261
Vs. HDR	0.919	0.339	2.490	0.868	0.679	0.244	1.889	0.458
Initial PSA	1.231	1.056	1.435	*0.008*	1.244	1.067	1.452	*0.005*
Duration of ADT	0.925	0.828	1.034	0.169	0.926	0.829	1.034	0.170
Age at RT start	0.982	0.942	1.023	0.379	0.972	0.931	1.015	0.198

*ADT* androgen deprivation therapy, *RT* radiotherapy, *CF* conventional fractionation, *HF* moderate hypofractionation, *LDR* low-dose-rate brachytherapy, *HDR* high-dose-rate brachytherapy

Significant findings are highlighted in italic

**Table 5:** Uni- and multivariable analysis of patients with intermediate-risk prostate cancer regarding biochemical control

Intermediate risk	Univariable analysis				Multivariable analysis			
Parameter	HR	95% CI (lower bound)	95% CI (upper bound)	*p*-value	HR	95% CI (lower bound)	95% CI (upper bound)	*p*-value
*Type of therapy (CF baseline)*								
Vs. HF	0.647	0.249	1.679	0.370	0.705	0.268	1.850	0.478
Vs. LDR	1.088	0.656	1.803	0.744	1.171	0.680	2.019	0.569
Vs. HDR	1.172	0.579	2.371	0.659	1.099	0.511	2.364	0.809
Gleason Score ≤ 6 vs. 7	0.837	0.533	1.314	0.440	1.203	0.612	2.365	0.591
T category 1a-c+ 2a vs. 2b+c	1.522	0.957	2.422	0.076	1.769	1.047	2.986	*0.033*
Initial PSA	1.020	0.965	1.079	0.480	1.053	0.968	1.146	0.232
Duration of ADT	1.004	0.990	1.018	0.613	1.004	0.989	1.019	0.637
Age at RT start	0.975	0.945	1.007	0.123	0.975	0.942	1.010	0.159

*ADT* androgen deprivation therapy, *RT* radiotherapy, *CF* conventional fractionation, *HF* moderate hypofractionation, *LDR* low-dose-rate brachytherapy, *HDR* high-dose-rate brachytherapy

Significant findings are highlighted in italic

The distribution of maximum late toxicities is displayed in Table 6. Overall, we observed 2 RTOG grade 4 GI toxicities, one in the CF and one in the LDR-BT group. These consisted of a colovesical fistula and the need for a permanent colo- and urostoma in the patient treated with LDR. However, this patient was previously irradiated with 5 × 5 Gy due to rectal cancer. The patient treated with CF required a transient colostoma due to an abscess with a fistula. Besides, we observed 7 RTOG grade 4 GU toxicities, one in the CF and 6 in the LDR-BT group. These consisted of an artificial bladder sphincter in the patient treated with CF. In the LDR-group, we observed the aforementioned urostoma, 2 TUR-Ps, and 3 catheter implantations. The highest rate of grade ≥ 2 GU toxicity was found in patients receiving LDR-BT after 3 months (63%) and was mainly due to the continuous use of tamsulosin, which was routinely prescribed after every LDR-BT. For HDR-BT, no 3‑month toxicity data were available. Besides, tamsulosin was not routinely prescribed in the HDR group.

**Table 6:** Maximum late toxicity in RTOG grades after treatment with conventional fractionation (CF), moderate hypofractionation (HF), or LDR- or HDR-brachytherapy

Gastrointestinal	CF (in %)	HF (in %)	LDR (in %)	HDR (in %)
4	0.3	0.0	0.2	0.0
3	0.8	3.6	0.2	0.0
2	21.8	19.4	8.7	0.6
1	16.3	11.5	19.3	1.9
0	60.9	65.5	71.5	97.5
*Genitourinary*				
4	0.0	0.0	1.0	0.0
3	4.5	2.0	2.7	0.6
2	20.1	25.0	66.9	18.0
1	21.8	21.0	18.1	8.1
0	53.6	52.0	11.2	73.3

**Fig. 2:**
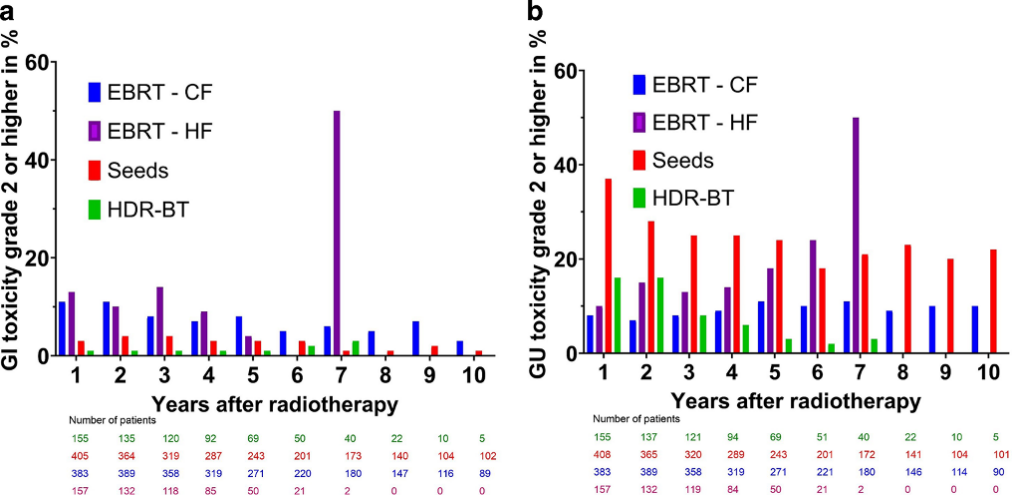
**a** Distribution of gastrointestinal toxicities using RTOG grading in percentages and numbers of patients over time after treatment with conventional fractionation (CF), moderate hypofractionation (HF), or LDR (seeds)- or HDR-brachytherapy. For each time point, the order from left to right is CF, HF, LDR-BT (seeds), and HDR-BT, **b** Distribution of genitourinary toxicities using RTOG grades in percentages and numbers of patients over time after treatment with either conventional fractionation (CF), moderate hypofractionation (HF), or LDR (seeds)- or HDR-brachytherapy. For each time point, the order from left to right is CF, HF, LDR-BT (seeds), and HDR-BT

The prevalence of late toxicities between 12 and 120 months after radiotherapy with a grade of 0 or 1 compared with 2 or higher over time can be found in Fig. 2a, b. DVH data for organs at risk were available for HDR-BT and are displayed in Supplement 2.

## Discussion

There are many treatment options for localized prostate cancer, and all of them provide excellent overall survival [2]. It is therefore of the outmost importance that the delivered treatment leads to as little toxicity as possible. Hoffman et al. showed a slight advantage of radiotherapy compared with surgery in terms of GU toxicity [16], looking at EBRT and LDR-BT.

As for tumor control, we were able to reproduce the expected results, showing no significant differences between treatment types, and when looking at LR and IR separately. However, there was a tendency for improved BC in the HF group, possibly due to the slight dose escalation that was performed in this group. This is in line with the ASCENDE trials, which demonstrated benefits of dose escalation in patients with IR and high-risk prostate cancer [17].

For late toxicity, we were able to observe a very low rate of both GU and especially GI toxicity in the HDR group. There are several studies looking at the effects of HDR-mono-BT as a stand-alone [18] compared with stereotactic EBRT [19, 20] or to LDR and EBRT with and without an HDR boost [21]. In all of them, HDR toxicity rates were very low. Morton et al. [22] compared 19 Gy single fraction HDR-BT with 2 × 13.5 Gy and were able to display an advantage regarding BC in the 2‑fraction group, but did not find an advantage regarding toxicity. Corkum et al. did the same [23], and were also unable to find differences regarding toxicity. Assuming an α/β of 1.5 Gy, 1 × 19 Gy and 2 × 13.5 Gy are 111 and 115 Gy EQD2. Yamazaki et al. [24] compared different schedules and fractionations between 7 and 9 fractions and found the least toxicity, with reported grade 2 or higher comparable to what we observed, when using 7 × 6.5 Gy, which equals an EQD2 of 104 Gy and is close to the 108 Gy EQD2 used in our study, while the others, with higher doses, led to more toxicity. However, the shorter follow-up might also, at least partly, contribute to the observed lower rates of toxicity in patients treated with HDR.

This is especially important, as the NCCN guidelines recommend HDR-mono-brachytherapy with 2 × 13.5 Gy or 2 × 9.5 Gy twice a day [5], while the GEC-ESTRO ACROP prostate brachytherapy guidelines [25] and the German S3 guideline [26] do not recommend the routine use of HDR-mono-BT at all, therefore limiting access to a treatment with comparable tumor control and low toxicity. With these results and discussed points in mind, we strongly suggest a randomized study be conducted to further investigate HDR-BT compared with EBRT; it should also look at different fractionation schemes, as 3 × 10.5 Gy does seem to provide an excellent safety profile, to provide the required evidence.

Regarding the higher maximal GU toxicity in patients treated with LDR-BT, this is mostly due to the continued use of tamsulosin after 3 months, which was routinely prescribed for every patient treated with LDR-BT. As shown in Fig. 2a, toxicity reported by patients treated with LDR-BT declines over time. For patients treated with moderate HF, we observed high rates of toxicity after 7 years for both GI and GU toxicity. This is most likely due to the fact that there are only two patients left in this group, with one of them reporting toxicity. Besides, we were unable to observe major differences regarding toxicity in patients treated with EBRT, although the CF group was mostly treated with 3D-conformal radiotherapy and the HF group exclusively with IMRT or VMAT. With the CHHiP trial showing no relevant differences between CF and HF in patients treated with IMRT [27], it is unlikely that the fractionation scheme is the cause of negating the expected lower toxicity in patients treated with HF due to IMRT. With the FLAME trial showing no major differences after dose escalation in patients treated with IMRT or VMAT either [28], we suspect that we are unable to observe a major difference in GU toxicity due to the proximity of the prostate to bladder and urethra, whereas for GI toxicity the use of the rectal balloon might lead to similar toxicities, as it creates a close proximity of the anterior wall of the rectum to the prostate and increases the distance for the other parts, possibly evening out the advantages of IMRT.

However, although the observed toxicities were low across the board, it is important to note that Hamdy et al. [2] showed the oncological feasibility of active surveillance in localized prostate cancer compared to surgery and radiotherapy. Therefore, one has to keep in mind, that no treatment at all leads to the least toxicities.

Regarding strengths of our study, we are able to report the results of a large bicenter cohort comparison of four available radiooncological treatment modalities in low- and intermediate-risk prostate cancer, which, to our knowledge, is the first such study. Assessment of toxicities was performed according to the RTOG/EORTC criteria in both centers. Interobserver variability in terms of toxicity is an old problem in radiotherapy [29]. Besides, missing values after 3 months for patients treated with HDR-BT might, at least in part, explain the excellent HDR-BT results regarding GI and GU toxicities in this group. However, looking at the DVH data in Supplement 2, we observed very low rectal D1 cm^3^, D0.1 cm^3^, and V75%. Therefore, with all the aforementioned bias, we still consider the low GI rates plausible.

A major weakness of our study is the uneven distribution of treatment types by center, as only one center provided patient data for patients treated with EBRT. This might contribute to the differences in reported side effects by treatment type. However, all the senior physicians were trained in the same institution, potentially reducing the extent of this problem. Another point is the fact that patients were able to decide which treatment they wanted, assuming an anatomy allowing for BT and meeting requirements for anesthesia for BT, leading to a selection bias with healthier patients in the BT groups. Besides, the included patients were treated over a period of more than 20 years, leading to other potential biases, such as stage migration, for example changes regarding the classification of patients with a T2c-staged prostate cancer, or changing treatment practices.

## Conclusion

All treatment types provided excellent BC in both patients with low- and with favorable intermediate-risk prostate cancer and were well received regarding late GI and GU toxicity, with low rates of RTOG grade 3 or 4 GI and GU toxicities. Accordingly, they are a valid alternative to surgery in this patient collective. HDR-BT showed a very low rate of RTOG grade 2 toxicity or higher, especially for GI toxicity, but possibly in part due to reporting bias. Nevertheless, our toxicity results are promising and strongly suggest the further evaluation of HDR-BT as a monotherapy in low- and favorable intermediate-risk prostate cancer in prospectively randomized trials.

### Supplementary Information

Supplement 1 covers the fractionation schemes used.
Supplement 2 covers the DVH-data of HDR-brachytherapy. Doses in EQD2, α/β 1.5 Gy.
