# Supplementary material for: Radiotherapy in localized prostate cancer: a multicenter analysis evaluating tumor control and late toxicity after brachytherapy and external beam radiotherapy in 1293 patients
Source: Strahlenther Onkol. 2024 Mar 15;200(8):698–705. doi: 10.1007/s00066-024-02222-w (PMC11272802; doi:10.1007/s00066-024-02222-w)
Supplement: Supplementary file 1 — Supplement 1 covers the fractionation schemes used. Supplement 2 covers the DVH-data of HDR-brachytherapy. Doses in EQD2, α/β 1.5 Gy. [file 66_2024_2222_MOESM1_ESM.docx]

Supplement 1: Fractionation schemes used

| Treatment type | Total dose in Gy | Dose per fraction in Gy | EQD2, α/β 1.5 Gy | Number of patients |
| --- | --- | --- | --- | --- |
| CF | 74 | 2 | 74 | 277 |
|  | 74.8 | 2.2 | 79.1 | 5 |
|  | 76 | 2 | 76 | 24 |
|  | 78 | 2 | 78 | 164 |
| HF | 73 | 25*2.4+5*2.6 | 82.1 | 182 |
| LDR | 145 | - | - | 480 |
| HDR | 31.5 | 10.5 | 108 | 161 |

CF: conventional fractionation, HF: moderate hypofractionation, LDR: LDR-brachytherapy, HDR: HDR-brachytherapy, EQD2: equivalent dose in 2-Gy fractions.

Supplement 2: DVH-data of HDR-brachytherapy. Doses in EQD2, α/β 1.5 Gy

| Data point | Median (IQR) |
| --- | --- |
| Urethra D1 cm³ in Gy | 106.64 (104.09/109.50) |
| Urethra D0.1 cm³ in Gy | 114.04 (112.14/116.87) |
| Urethra D30% | 109.00 (106.64/111.40) |
| Urethra D10% | 112.34 (110.48/114.81) |
| Urethra V75% | 1.96 (1.74/2.21) |
| Urethra V115% | 0.07 (0.02/0.26) |
| Urethra V120% | 0.00 (0.00/0.00) |
| Rectum D2 cm³ in Gy | 53.18 (48.31/58.10) |
| Rectum D1 cm³ in Gy | 64.63 (59.93/67.70) |
| Rectum D0.1 cm³ in Gy | 78.49 (74.97/79.75) |
| Rectum D10% | 71.85 (67.33/76.11) |
| Rectum V75% | 0.25 (0.10/0.38) |
